# Exploring sleep outcomes in youth across settings: Are there differences based on rurality or medically underserved status in the ECHO cohort?

**DOI:** 10.1016/j.sleep.2025.108754

**Published:** 2025-12-27

**Authors:** Brittany D. Lancaster, Anna Wallisch, Emily A. Knapp, Xuan Li, Lacey A. McCormack, Kelly A. Hirko, Traci A. Bekelman, Christine W. Hockett

**Affiliations:** a Department of Psychology, Mississippi State University, MS, P.O. Box 6161, Mississippi State, MS, 39762, USA; b Division of Developmental and Behavioral Sciences, Department of Pediatrics, University of Kansas Medical Center, 2000 W. Olathe Blvd, Mailstop 4004, Kansas City, KS, 66160, USA; c Department of Epidemiology, Johns Hopkins Bloomberg School of Public Health, 700 E Pratt Street, Baltimore, MD, 21202, USA; d Avera Research Institute, Avera McKennan Hospital & University Health Center, 6001 S Sharon Ave, Suite 2, Sioux Falls, SD, 57106, USA; e Department of Pediatrics, University of South Dakota School of Medicine, 1400 W 22nd St, Sioux Falls, SD, 57105, USA; f Department of Epidemiology & Biostatistics, College of Human Medicine, Michigan State University, 909 Wilson Road, Room B601, East Lansing, MI, 48824, USA; g Lifecourse Epidemiology of Adiposity and Diabetes (LEAD) Center, University of Colorado Anschutz Medical Campus, 1890 N. Revere Court, Aurora, CO, 80045, USA

**Keywords:** Sleep, Rural, Urban, Medically underserved, Children, Environmental influences on child health, outcomes

## Abstract

**Objective:**

To evaluate associations between rurality and medically underserved status with sleep outcomes (e.g., duration, bed and wake times, latency) among youth in the United States.

**Methods:**

The study sample included 22,234 youth (aged 1–17 years) from the Environmental influences on Child Health Outcomes (ECHO) Program. Sleep was measured by parent-report questionnaire for children ages 7 and under and child-report for older children. Residential addresses and Federal Information Processing Standards codes were utilized to classify participants into two binary exposure variables: rural vs. nonrural and medically underserved vs. not medically underserved. Differences between groups for each developmental period were compared using t-tests and chi-squared tests.

**Results:**

Across children ages 1–12, rural youth went to bed significantly earlier (mean differences [MD] range from 18 to 52 min) and woke earlier (MD range 15–52 min) compared to non-rural youth. Children aged 1–12 living in medically underserved areas were significantly less likely to meet sleep recommendations (MD range 3.2 %–6.6 % less), and youth of all ages tended to have later bedtimes (MD range 7–30 min) compared to youth not living in medically underserved areas.

**Discussion:**

This study is novel because it evaluates differences in youth sleep health based on rurality and medically underserved status in the United States. Multiple variations in children’s sleep health were identified based on living in medically underserved areas while minimal differences were noted based on rurality. Future research should focus on the development or dissemination of effective programs into pre-established resources (e.g., daycares, community centers) for youth in medically underserved areas.

## 1. Introduction

Health disparities persist across the United States, particularly for populations residing in rural [1,2] and medically underserved areas (MUAs) [3,4]. Individuals in rural communities often face unique barriers to healthcare access [5], including provider shortages, transportation difficulties, and limited health resources, which can contribute to poorer health outcomes compared to their non-rural counterparts. Similarly, those living in MUAs, geographic areas with a lack of access to primary care services or areas facing additional barriers to healthcare access [6,7], —regardless of rural or urban status-—experience reduced access to preventive care and treatment, exacerbating existing health inequities. MUA status, broadly, is a composite variable that allows identification of regions where health care systems may be overburdened or under-resourced. These conditions can indirectly impact children’s access to health-promoting services and support, including those relevant to sleep. While rurality and medical underservice frequently overlap, they are distinct constructs with potentially different impacts on health (e.g., rurality impacted stage of cancer diagnosis regardless of MUA status [8], those in rural areas had higher odds of obesity, but not those in MUAs [9]). Despite evaluations of how other health behaviors (e.g., physical activity [10,11] and nutrition [10,11]) may differ for youth in rural communities compared to urban youth, no research has evaluated how living in rural areas or MUAs may affect youth sleep health or sleep ecology (i.e., contextual factors affecting sleep [12]) within the United States.

Achieving adequate sleep among youth is crucial as it is associated with higher quality of life, greater emotional regulation, better academic achievement, and lower adiposity [13]. In rural communities specifically, deficits in youth sleep duration have been linked to adverse impacts on mental health [14] and health risk behaviors (e.g., smoking, alcohol, drug use) [15]. Disparities in youth sleep [16,17] may be best understood through a socio-ecological framework [18] in which multiple neighborhood and community factors can impact youth sleep. Examples include limited access to sleep or behavioral healthcare, longer commute times to daycare, school or work affecting time in and out of bed, variations in school start times, and noise pollution. Without making comparisons to youth in urban settings, prior research has shown that approximately 40 % of adolescents living in in the rural Appalachian region of North Carolina did not obtain at least 6 h of sleep on a typical school night (notably lower than the recommended 8–10 h) [14].

Most of the research on differences in sleep health across rural and non-rural youth is based on data from outside the United States, and findings are inconsistent. For example, rural Indian adolescents had notably shorter sleep duration [19] and more daytime sleepiness [20] compared to their urban counterparts. However, other studies found no differences in sleep duration or bed- and wake-time between groups [21–24] or that adolescents had shorter sleep duration and later bedtimes in urban compared to rural areas [25–28]. These varying results may be a function of how rural environments differ across countries with regards to access to technology, environmental noise, school workloads [29] and access to electric lighting [30]. Of note, the majority of these comparisons between rural and non-rural populations have focused on adolescents. Thus, an examination of how rurality may impact United States residents, across various age groups, is warranted.

Similarly, no research has specifically examined whether living in MUAs is associated with youth’s sleep health. The literature has consistently demonstrated that closely-related constructs, such as socioeconomic status (SES), may impact youth sleep [31–33]. Specifically, youth in families with lower SES have a shorter sleep duration [31,32], delayed bedtimes [32,33], lower sleep quality [31], and greater daytime sleepiness [31]. Research has also demonstrated that SES may impact sleep ecology, with results suggesting that lower income and lower education families are less likely to implement a bedtime routine [34] and more likely to have bedroom screentime [35]. Given that MUAs are defined based in part on poverty rates, these findings suggest that sleep disparities in MUAs with additional health care access barriers may be even more pronounced.

Characterizing differences in sleep and sleep ecology within the United States’ MUAs or rural communities allows us to identify entire communities with elevated, population-level needs, allowing for macro-level changes (e.g., policy changes, community-level interventions) to help improve population health. Identified differences can also highlight a need for existing federal programs, such as the National Institute of Health’s Institutional Development Award (IDeA) States Pediatric Clinical Trials Network (ISPCTN) who focus on rural and MUAs to support the implementation and evaluation of pediatric sleep interventions in these high-need populations.

Thus, this study aims to describe associations between rurality and medically underserved status with sleep health across childhood and adolescence in the United States. This study’s primary outcomes include sleep duration, bed and wake times, adherence to sleep recommendations, and sleep latency. It was hypothesized that both youth in MUAs and rural communities would differ in their sleep outcomes (e.g., shorter sleep duration, less likely to meet sleep recommendations) compared to youth not living in these areas. As an exploratory secondary aim, we additionally evaluate sleep ecology, the environmental and behavioral factors that influence a child’s sleep patterns. The results of this study may inform targeted efforts to improve sleep outcomes and highlight specific dimensions of sleep health that may benefit from intervention. Even if findings are null, these comparisons can establish a foundation for future research to evaluate family-level resilience factors or successful programs and policies that support children’s sleep health in vulnerable geographic areas.

## 2. Methods

### 2.1. Study design and participants

The Environmental influences on Child Health Outcomes (ECHO) Program [36] includes a national consortium of 69 pregnancy and pediatric cohort study sites across the United States, including Puerto Rico. Since 2019, the ECHO Cohort has used a common data collection protocol to follow pregnant persons and children recruited from clinic- and community-based settings to investigate how early life exposures shape respiratory, obesity, neurodevelopment, positive health, and pre-, peri-, and post-natal outcomes.

**Fig. 1.**
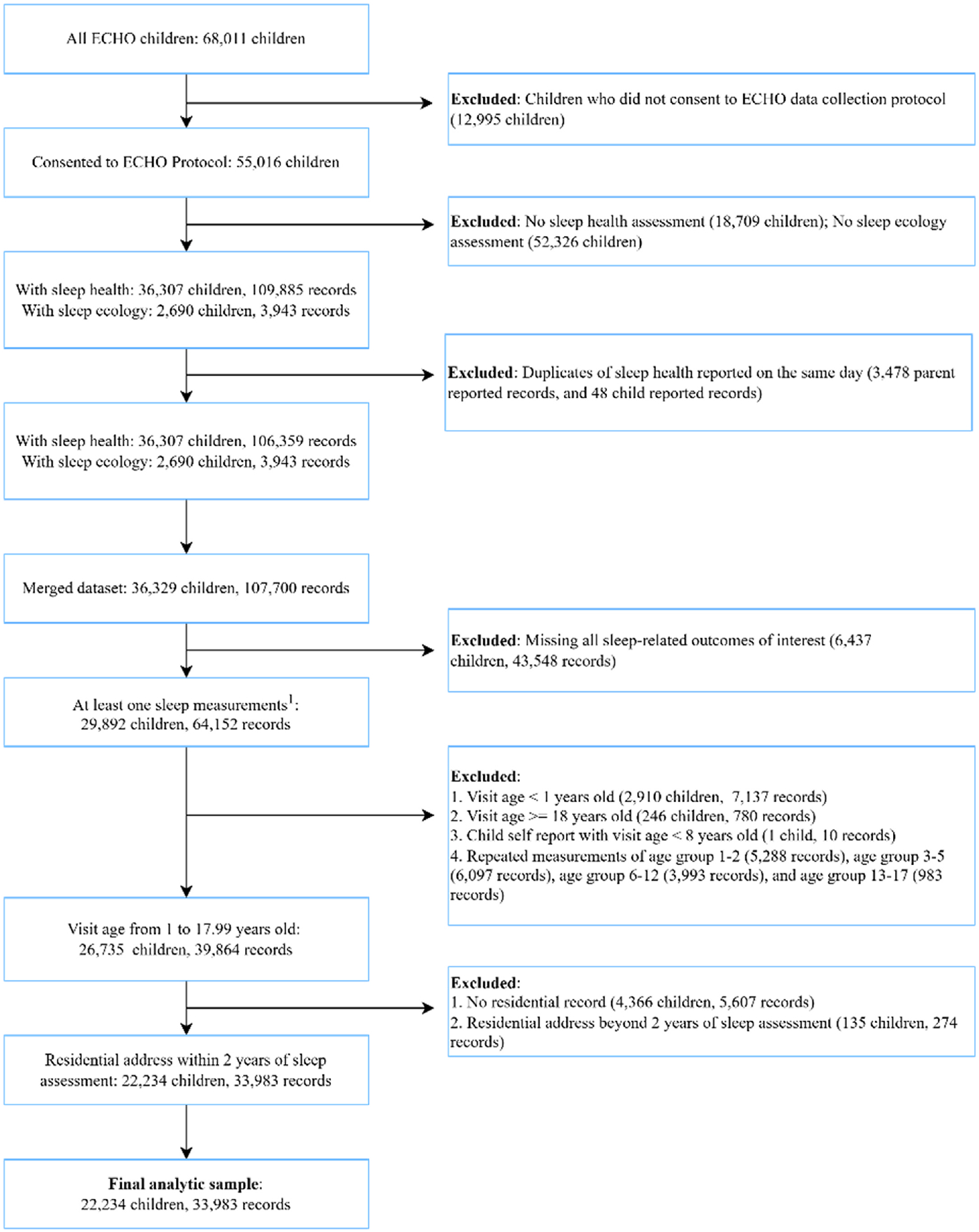
Study inclusion and exclusion criteria.

Child participants who consented to data sharing were included in this analysis if they or a parent/caregiver proxy reported at least one sleep outcome between the ages of 1–17 years old and a residential address within two years of the sleep assessment (see Fig. 1). The study protocol was approved by local institutional review boards (IRBs) and/or the single ECHO IRB, WIRB (Western Institutional Review Board)-Copernicus Group (WCG). Written informed consent or parent’s/guardian’s permission was obtained along with child assent as appropriate.

### 2.2. Outcomes

Primary outcomes of interest include sleep duration (both daytime naps and nighttime duration), wake time and bedtime (weekday and weekend), and sleep latency, defined as the time taken to fall asleep. We also assessed the likelihood that children met age-specific sleep recommendations (11 h or more for children aged 1–2 years, 10 h or more for children 3–5 years, 9 h or more for children 6–12 years, and 8 h or more for children 13–17 years) [37]. Sleep was measured by parent-report questionnaire for all children under eight years of age, and children 8 or older could self-report [38,39]. As an example, the parent-report sleep health questionnaire is provided in Supplemental File A. The questionnaires queried sleep behaviors during the previous 7 days. In addition to standardized data collection forms developed for the ECHO protocol, cohort study sites also contributed sleep data collected prior to the implementation of the ECHO protocol; these data are also included in this analysis. Included data was assessed between 2004 and 2023.

Sleep ecology is an important component of youth sleep as it often represents the malleable behaviors possible to target with sleep interventions [40,41]. Sleep ecology was assessed in this study by evaluating the use of electronic devices before falling asleep, bedtime routine, and consistency of wake time and bedtime. Each of these variables has been previously linked to child sleep duration and sleep timing [42–44]. These outcomes were reported by the parent (1–7 years) and the child could self-report (8 years and older). Ecology was included as a secondary outcome, as these data elements were only collected in a sub-sample of ECHO Cohort participants.

### 2.3. Exposures

Participants or their parents reported residential addresses as well as the dates living at each address. Using this information, addresses were geocoded, and Federal Information Processing Standards (FIPS codes) for the census tract of residence were extracted for the calendar year of the sleep outcome. Using FIPS codes, participants were classified based on two separate binary exposure variables: (1) rural vs. non rural, and (2) MUA vs. not MUA. Rurality was defined using Rural-Urban Continuum Codes (RUCC): a RUCC of 1–3 was considered non-rural, and 4–9 was considered rural [45]. Medically underserved areas were defined using MUA designations from the Health Resources & Services Administration (Index of Medical Underservice Score of less than or equal to 62.0 indicates a MUA, scores range from 0 to 100) [6]. MUAs are areas that do not have sufficient access to primary health care services, and designations are based on the ratio of providers per 1000 population, the percent of the population at 100 % of the federal poverty level, the percent of the population age 65 and older, and the infant mortality rate [7]. While not all components are likely directly linked to child sleep, the designation serves as a proxy for regions where health care systems may be overburdened or under-resourced—conditions that can indirectly impact children’s access to health-promoting services and support, including those relevant to sleep.

### 2.4. Demographics

We reported the following demographic characteristics of children: child sex, child race, and child ethnicity. We also included socioeconomic characteristics of the child’s household, including educational attainment of the mother and total combined income of the household. Additionally, we report the following characteristics of the census tract of residence: the percentage of people age 25 years or older with no high school diploma and the socioeconomic status score of the Social Vulnerability Index (scores range from 0 to 1.0, with higher scores indicating higher socioeconomic vulnerability in the participant’s community) [46].

### 2.5. Statistical analysis

Children were grouped by age (1–2 years, 3–5 years, 6–12 years, and 13–17 years). We separated the age groups based on established youth sleep recommendations [37]. For older age groups (6–12 years and 13–17 years), parent-report and self-report of sleep outcomes are reported separately. Children may have an observation in multiple age groups, and/or in both parent and self-report columns. Within each age and parent or self-report group, we selected the earliest sleep outcome for inclusion in the analytic sample if multiple were present. Self-report observations for children under age 8 were excluded. During exploratory data analysis, we noticed a higher-than-expected prevalence of weekend bedtime of 12:00 (i.e., 12:00pm). These observations were set to 00:00 (i.e., 12:00am). To exclude implausible values for sleep, we set observations to missing when total nighttime sleep was less than 4 h or greater than 20 h, wake time before 4:00 or after 15:00, naptime of greater than 5 h for children 1–2 years and greater than 4 h for all other age groups, or bedtime earlier than 17:00pm or after 02:00 (weekday) or 03:00 (weekend). Additionally for toddlers and preschoolers, we estimated total daily sleep duration (nighttime plus naps) because sleep recommendations at these ages include naps. Nap duration was calculated by multiplying the reported number of days the child napped per week (response categories: none = 0; 1 day = 1; 2–3 days = 3; 4–5 days = 5; 6–7 days = 7) by the usual nap length, then dividing by 7 to obtain an average nap length per day. This value was added to reported nighttime sleep duration to estimate total sleep. Because nap frequency was assessed categorically and required conversion to approximate days, this measure is an estimate rather than a directly reported value.

We present mean and standard deviations for continuous variables and number and percent for categorical outcomes. Differences in the mean (for continuous variables) between children living in rural and non-rural areas and medically underserved and non-medically underserved areas, respectively, were compared using t-tests. Differences in the proportions of categorical variables were compared using chi-squared tests. Statistical significance was defined as p < .05.

### 2.6. Data availability statement

Select de-identified data from the ECHO Program are available through the National Institute of Child Health and Human Development’s (NICHD’s) Data and Specimen Hub (DASH). Information on study data not available on DASH, such as some Indigenous datasets, can be found on the ECHO study DASH webpage.

## 3. Results

**Fig. 2.**
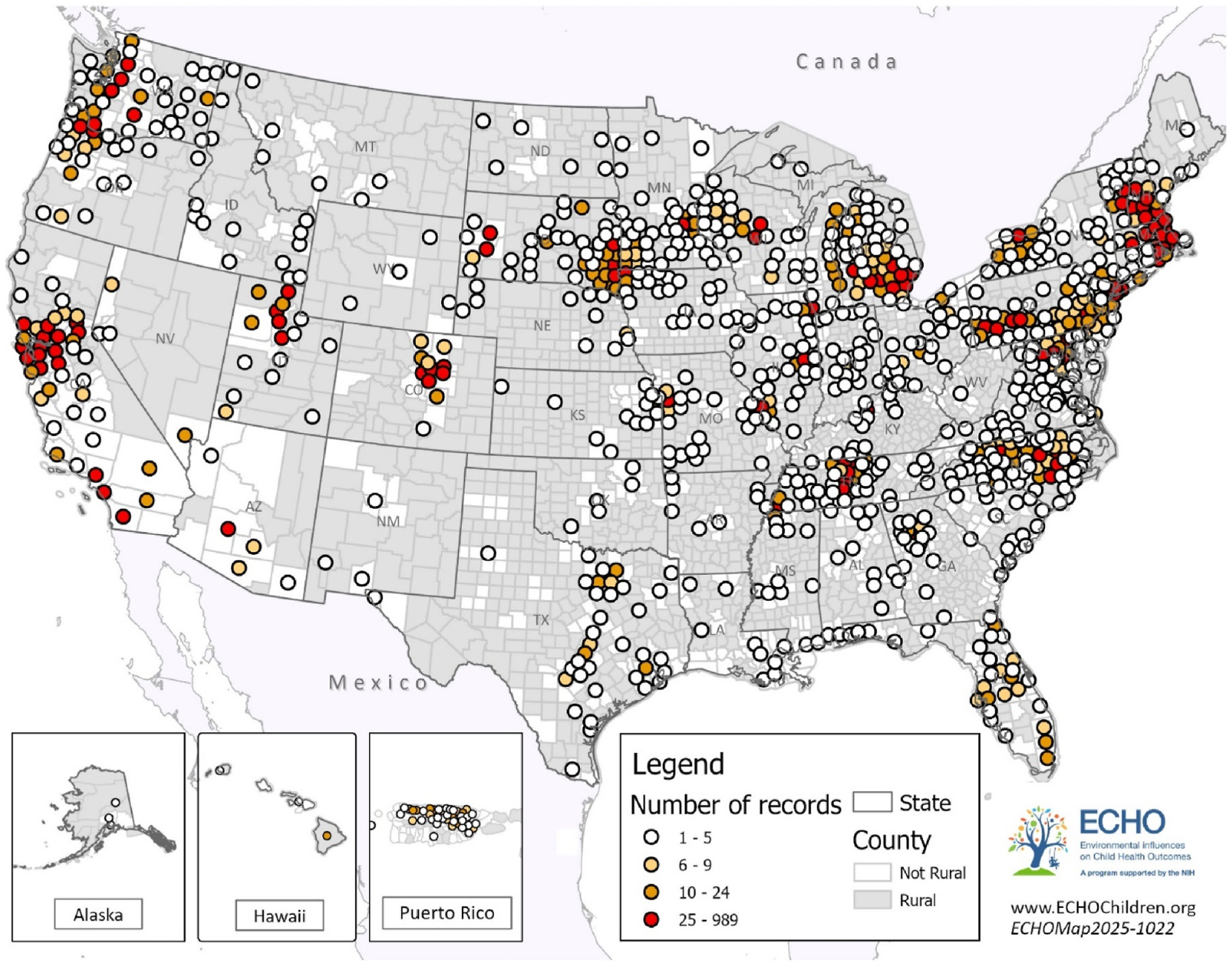
Map of participants’ residence, by rural status. Caption: Fig. 2 shows the county of residence for participants in the study sample. Grey shading indicates rural counties (defined for this study as USDA’s Rural-Urban Continuum Codes (RUCC) of 4–9; non-rural counties defined as RUCC 1–3).

**Fig. 3.**
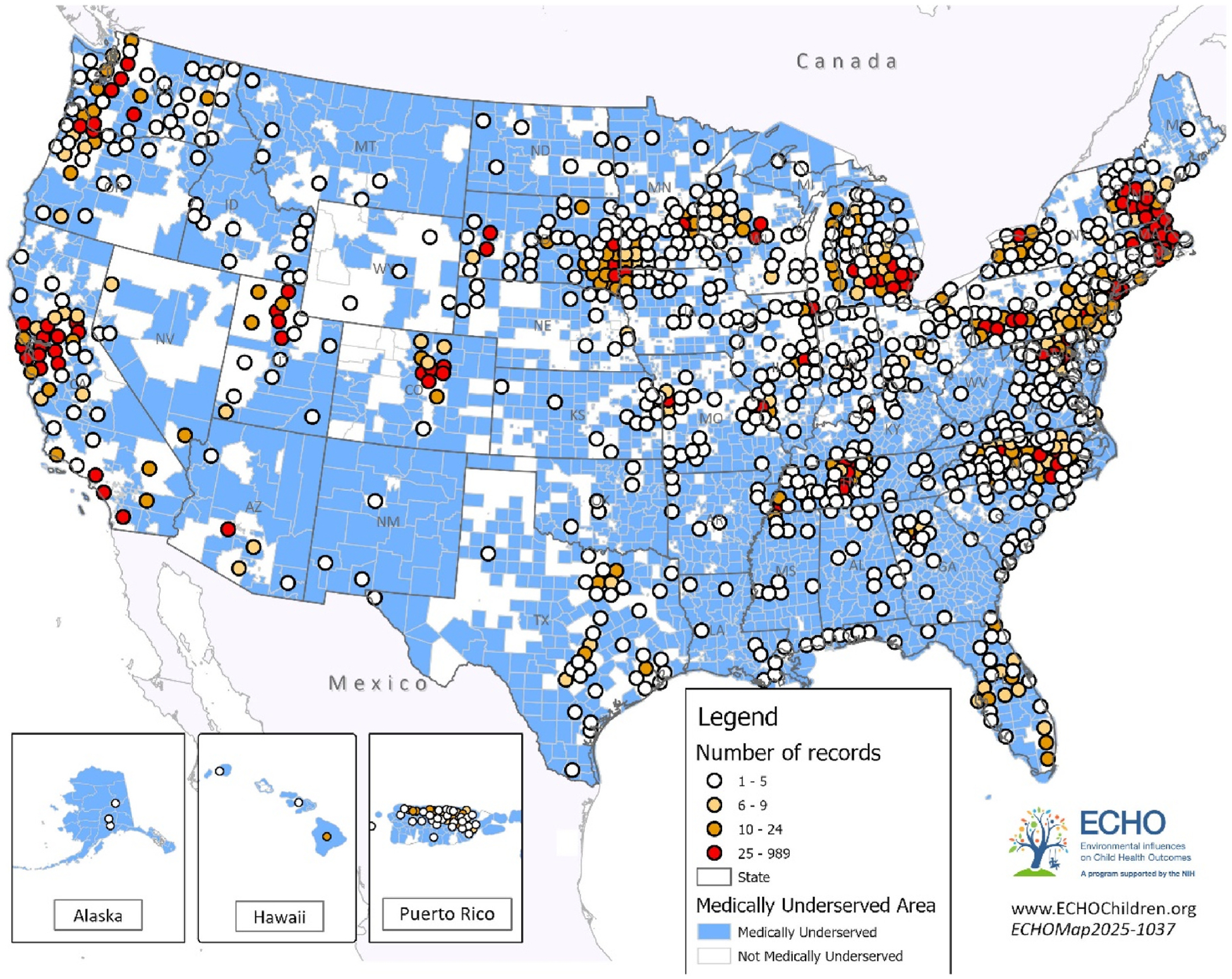
Map of participants’ residence, by medically underserved status. Caption: Fig. 3 shows the county of residence for participants in the study sample. Blue shading indicates medically underserved areas which were defined using medically underserved designations from the Health Resources & Services Administration (Index of Medical Underservice Score of less than or equal to 62.0 indicates a medically underserved area, scores range from 0 to 100).

The study sample included 22,234 children from 51 cohort sites (Fig. 2) and was grouped into the following categories: toddlers (ages 1–2 years, *n* = 9685), preschoolers (ages 3–5 years, *n* = 10,943), school-age children (ages 6–12 years, *n* = 9108) and adolescents (ages 13–17 years, *n* = 2750). Table 1 displays the demographic characteristics of the overall sample as well as stratified by age group. The majority of children were White race (61.7 %), 16.9 % were Black race, 10.5 % reported multiple races, 4.1 % were Asian, and 1.7 % were American Indian; 23.6 % were Hispanic ethnicity. Half of children in this sample had mothers with a bachelor’s degree or higher. The average Social Vulnerability Index SES score is 0.4 [47], which is below the national median (indicating lower SES vulnerability). Of the 22,234 youth in our sample, 6976 lived in MUAs, 908 of whom also lived in rural areas (13 %). Of the 1853 youth living in rural communities, 945 (51 %) did not live in an MUA. Figs. 2 and 3 depict the geographic distribution of participants across rural and non-rural counties and across MUAs and non-MUAs, respectively, illustrating where study participants resided.

Comparisons between rural and non-rural and MUA versus non-MUA are presented by age group in Tables 2–5. Primary outcomes and significant differences are highlighted below in text. Missing data was variable across age groups and sleep outcome; each table provides missing data counts. Further, given that sleep ecology was only collected in a sub-sample of ECHO participants, there were minimal sleep ecology reports from rural youth. Given this, only differences in sleep health for MUA versus non-MUA were evaluated.

### 3.1. Toddler comparisons

Table 2 summarizes between group comparisons for toddlers. Average total 24-h sleep duration did not differ between toddlers in rural (12.6 h) and non-rural (12.7 h) areas. One two-year-olds living in rural areas had earlier wake times (all *p* < .001) and bedtimes (all *p* < .001) on both weekdays and weekends: waking at an average time of 06:58 (SD 56 min) and going to bed at 20:05 (SD 55 min) on weekdays compared to 07:22 (SD 70 min) and 20:37 (SD 70 min) in non-rural counties. Parents reported that children fell asleep faster in rural compared to non-rural areas (18.6 min compared to 22.6 min, *p* < .001) and napped for longer (122.1 compared to 115 min, *p* = .005). Toddlers living in MUAs were less likely to meet sleep recommendations [37] (82.0 % vs 91.4 %, *p* < .001) and slept significantly less in a 24 h period compared to toddlers in non-MUAs (12.3 h compared to 12.9 h; *p* < .001). Children living in MUAs had later wake times (all *p* < .001) and bedtimes (all *p* < .001) on both weekdays and weekends, for example, weekday bedtime at 20:56 compared to 20:26 in non-MUAs. Children in MUAs slept for fewer hours per night (10.8 h compared to 11, *p* = .002) and had about 10 min longer average nap times (122.1 min compared to 112.4 min, *p* < .001). Children living in MUAs were less likely to nap (*p* < .001), however.

### 3.2. Preschooler comparisons

Table 3 reports between group comparisons for preschoolers. Parents of preschool children reported a mean total nighttime sleep of 10.9 h across rural, non-rural, MUAs, and non-MUAs. When comparing total 24-h sleep duration (naps + nighttime sleep), youth in rural areas obtained less sleep (11.3 h) compared to non-rural youth (11.7 h; *p* < .001) and were thus less likely to meet the 24-h sleep recommendations (85.6 % versus 94.5 %; p < .001). This is largely driven by non-rural populations having an increased frequency of naps (p < .001). Similar to toddlers, 3–5-year-old children in rural areas had earlier wake (all *p* < .001) and bedtimes (all *p* < .001) on both weekdays and weekends. Non-rural children in this age group had weekend bedtimes almost an hour later than rural children (21:10 vs 20:18). There were no significant differences in the time to fall asleep for rural preschoolers compared to nonrural.

Preschoolers living in MUAs were less likely to meet sleep recommendations [37] (91.7 % vs 94.5 %, *p* = .012). Preschoolers living in MUAs had later wake times (all *p* < .001) and bedtimes (all *p* < .001), for example weekday bedtimes of 20:51 (SD 62 min) compared to 20:35 (SD 56 min) in non-MUAs. Preschoolers in MUAs napped longer (96.4 min compared to 89.9, *p* < .001) and were more likely to nap most days of the week (*p* < .001). The proportion of preschoolers frequently delayed bedtime was slightly higher in non-MUA vs. MUA (17.1 % vs. 11.6 %, *p* < .001) and on average took longer to fall asleep (29.4 min compared to 25.1, (*p* < .001).

### 3.3. School-age children comparisons

Table 4 reports sleep outcomes reported by the parents of school-age children ages 6–12 years (self-reported sleep outcomes for children 8–12 are presented in Supplemental Table B). Parents of school-age children reported a mean total nighttime sleep of 10.2 h across rural, non-rural, MUAs, and non-MUAs. As observed in younger children, 6–12-year-olds living in rural communities had earlier wake (15 min on weekdays, 28 min on weekends, all *p* < .001) and bedtimes (18 min on weekdays, 27 min on weekends, all *p* < .001). Napping more than one day a week was uncommon at this age, though reported naptime was longer in non-rural children (94.6 min compared to 82.8 min, *p* = .037). There was no difference in time to fall asleep based on rurality. The proportion of school-age children meeting sleep recommendations did not differ by rurality, though school-aged children living in MUA were less likely to meet sleep recommendations [37] (92.2 % vs 95.4 %, *p* < .001). Parents of children in MUAs reported similar child wake time on weekdays (07:02 and 06:59), and later bedtimes on weekdays (20:53 and 20:46, respectively, *p* < .001). On weekends, school-age youth in MUAs had significantly later bed (21:54 compared to 21:34, *p* < .001) and wake times (8:21 compared to 7:56, *p* < .001). Children living in MUAs were less likely to delay bedtime (15.7 % almost always or always compared to 19.2 % in non-MUAs *p* = .002) and took longer to fall asleep (29 min compared to 26.1, *p* < .001).

### 3.4. Adolescent comparisons

Among adolescents (ages 13–17 years), self-reported sleep duration, waketime on weekdays or weekend, bedtime on weekdays, and sleep latency did not differ by rurality (Table 5). Non-rural teens reported a later weekend bedtime of 23:59 compared to 23:38 for rural teens, *p* < .001. Similar patterns were seen in parent-reported data (Supplemental Table C). Adolescents in MUAs reported longer sleep duration (average of 9.8 h compared to 9.5 h, *p* = .009), later wake *(all p* < .001) and bedtimes (all *p* < .01) on both weekdays and weekends, and a longer sleep latency (39.2 min compared to 34.8, *p* = .011). There was no significant difference in the proportion of adolescents meeting sleep recommendations [37] by MUA status.

### 3.5. Sleep ecology outcomes

Sleep ecology outcomes among children living in MUAs compared to non-MUAs are presented, by age group, in Supplement Tables D–G. Toddlers in non-MUAs were less likely to report always following a bedtime routine and trying to fall asleep at the same time each night compared to those in MUAs. Parents of preschoolers living in MUAs were more likely to report their child played video or computer games or watched TV before bedtime. No differences in sleep ecology outcomes were observed for 6–12-year-olds. Adolescents in non-MUAs were more likely to follow a bedtime routine but otherwise sleep ecology outcomes did not differ between MUAs and non-MUAs.

## 4. Discussion

This study is the first to evaluate differences in child sleep outcomes based on rurality and MUA status in the United States. Examining these groups separately allows for a more nuanced understanding of which geographic and systemic healthcare differences shape sleep health. Highlighting the importance of separately evaluating these populations, this study found that rural and MUA groups varied from their counterparts in different ways. There were more consistent differences in sleep outcomes for MUA populations (e.g., more likely to have not met sleep recommendations, later bedtimes), while fewer differences emerged between rural and non-rural groups.

More specifically, children and adolescents living in MUAs had later bed and wake times, and subtle differences in sleep duration compared to those residing in non-MUAs. While our study is the first assess youth sleep differences in MUAs, our findings generally align with results from prior systematic reviews [31,32] demonstrating shorter sleep duration and later bedtimes associated with lower SES, with stronger associations in earlier childhood. Compared to those in non-MUAs, shorter sleep duration was observed for toddlers in MUAs while sleep duration was longer for older adolescents in MUAs; although differences were minimal and may not be clinically meaningful. Across all age groups, youth residing in MUAs had later bedtimes and wake times and took a slightly longer time to fall asleep (range 2.9–4.6 min longer) compared to those in non-MUAs. Interestingly, preschoolers and school-age children in MUAs were less likely to report delaying bedtimes compared to those in non-MUAs. This may suggest that the later bedtimes in this age group reduce resistance to going to sleep and may highlight a need to provide parents with additional tools to implement earlier bedtimes and reduce bedtime resistance with younger youth.

Notably, differences by MUA status for most sleep outcomes were more pronounced in early childhood. For example, the percentage of youth meeting sleep recommendations was lower in MUAs for toddlers, preschoolers, and school-age children, but did not differ among adolescents. Differences in sleep patterns as children age, such as increasingly self-regulated sleep patterns, may introduce variability in sleep outcomes among older adolescents. For example, biological and social factors during the transition to adolescence, including changes in school start times, and extracurricular demands, would likely impact those in both MUA and non-MUAs, and may partially explain the later bedtimes and lower sleep duration observed among adolescents regardless of residence in MUAs in this study.

This study also examined differences in sleep ecology between youth living in MUAs versus not. Sleep ecology is an important construct to evaluate as it is often a target for improving sleep onset and duration in children and adolescents [40,41]. Our study found few differences in sleep ecology between MUAs and non-MUAs. Of the observed differences, toddlers and adolescents in non-MUAs followed a bedtime routine more frequently compared to toddlers and adolescents in MUAs. Following a bedtime routine has numerous benefits for both toddlers [44] and adolescents [48]; however, factors like parental fatigue [49], maternal depression [50], variable work schedules [51], single parent households [52], and food and housing insecurity [50], which are likely more prevalent among medically underserved families, can lead to inconsistent bedtime routines. Whereas, for preschoolers in non-MUAs, technology/electronics were used less frequently before bedtime compared to those living in MUAs. Our finding aligns with other literature that also suggests that low-income families and parents with lower education levels use screen time more frequently compared to higher income and parents with a higher education level [53]. Screen time in preschoolers is associated with difficulty falling asleep, increased tiredness [53], and decreased sleep duration [54]. Interestingly, this finding was not observed in any other age group. For adolescents, rates of screen use before bedtime were high across both MUA and non-MUA groups, suggesting that this behavior is common regardless of geographic context. For school age youth and toddlers, the limited sample size of youth living in MUAs for sleep ecology outcomes may have limited our power to detect effects. Overall, our findings suggest there may be an increased need to support families in MUAs with practical strategies for bedtime routines and reducing screen time before bed to support better sleep duration and quality.

With respect to comparisons between rural and non-rural populations, there were no differences in total sleep duration or meeting sleep recommendations between rural and non-rural children for toddlers, school-age youth, or adolescents. However, there was a significant difference for preschoolers in 24-h sleep duration and likelihood of meeting sleep recommendations that appeared to be largely explained by non-rural youth having an increased frequency of naps. These findings may suggest that there are additional barriers to rural preschoolers obtaining naps that should be further explored in future research.

Previous studies comparing sleep health among rural versus nonrural children in the United States are limited, with most evidence coming from other countries. Consistent with the findings reported here, nighttime sleep duration did not differ between urban and rural children and adolescents in some industrialized countries (e.g., Japan, Germany) [23,55]. However, studies in Canada, South Korea, and China demonstrated a longer sleep duration in rural compared to non-rural youth [27,28,56,57]. While most studies conducted outside the United States indicated a protective or null effect of rural residence, some studies have indicated that rural adolescents had worse sleep compared to their peers [19,20,29]. One study in Mexico found that rural adolescents had worse sleep efficiency (e.g., more time awake during their sleep cycle) than those in urban areas, and were able to identify that these findings may be explained by rural youth’s greater exposure to light and shared sleep spaces [29]. Thus, differences in the direction of the association across countries may be due to country-specific features of the rural environment or potentially differing cultural or religious practices affecting bedtime between rural and urban populations. Of note, our study could not evaluate sleep efficiency due to a lack of information regarding nighttime awakenings. Future examinations of sleep efficiency and quality across geographical areas in the United States may illuminate additional differences between rural and urban populations. Further, the majority of these studies were conducted among adolescents, highlighting a gap in our understanding of geographic differences in sleep among younger populations.

One consistent difference (from ages 1–12) was that children in rural communities generally woke up and went to bed earlier compared to non-rural children. Due to the high percentage of working parents [58] in rural areas and the distance [59] rural individuals may live from their place of employment and childcare, it is plausible that within this age group, children are being taken to daycare or school by working parents/caregivers who need to complete drop off prior to driving to and arriving at work, thus requiring an earlier wake time and, subsequently, an earlier bedtime. Likewise, the lack of difference between wake times on weekdays among rural and non-rural adolescents (aged 13–17 years) could be explained by less reliance on caregivers for transportation to school in the morning. Interestingly, studies outside the United States have demonstrated earlier chronotypes among rural versus urban adolescents [24,60] but this was not replicated in our sample. Overall, minimal differences were seen in sleep outcomes for rural and non-rural youth. This complements other literature showing comparable levels of other health behaviors, such as physical activity [61], across geographic contexts, despite some differences in how [62] or when these behaviors may occur.

This study has important implications to consider. Our findings indicate that children ages 1–12 years residing in MUAs are significantly less likely to meet current sleep recommendations. This may be attributed to the later bedtimes or slightly longer sleep latencies identified in this group. Fortunately, some previous interventions designed for children in lower SES environments have been effective at improving sleep duration [63,64]. However, not all approaches have been successful. For example, a recent sleep intervention delivered to lower SES families of preschoolers in a Head Start program [65] did not result in any clinically meaningful benefits for families. This underscores the need for future research to identify which intervention components are most effective and how they can be adapted for families in MUAs. Further, our results suggest that a focus on bedtimes routines and limiting screen time may be particularly helpful for toddlers and preschoolers in these communities. As previous research intervening on these factors has resulted in improved sleep for youth [40,41], future research can consider if integrating these evidence-based programs into resources (e.g., daycares, community centers, schools) within MUAs results in improvements. Given that rural youth tend to wake and go to bed earlier, providers working with children who are not getting sufficient sleep might consider leveraging this pattern by encouraging earlier evening routines. An additional implication of this research is that it is important to consider rural and MUA groups separately. At times, these groups may be considered as a single group [66] given the overlap in limited health care access; however, our study illuminates that these separate classifications (that do overlap) differentially predict health outcomes. As rurality and MUA status do overlap, some observed differences may reflect shared variance rather than the unique effect of either factor. Future analyses using multivariable models and interaction terms could help disentangle these influences.

Several limitations and potential sources of bias may have influenced our findings. The sleep domains and outcomes were measured via parent- and child-report measures. The use of subjective sleep data is known to present risk for bias. [67]. Additionally, we may have un-measured confounding, such as seasonal effects and changes in routines due to school calendars. We were unable to account for this, since at each site different school calendars were implemented. Further, missing data precluded analysis of sleep ecology differences in rural populations versus non-rural, additionally, the missing data may limit the generalizability of findings particularly for those included in the rural sample. This missing data is likely due to some data being collected before the sleep health questionnaire were introduced. In addition, relying solely on a binary rural/non-rural classification may limit our understanding of the complexities of rurality as a spectrum and the implications of that for child sleep. Future studies with larger rural samples should consider additional delineations of the data (e.g., remote areas, suburban) for a more thorough consideration of how geography may impact youth sleep. Importantly, more information about parent and child routines and daily activities would be helpful in contextualizing differences in wake times and bedtimes on weekdays and weekends between different populations (e.g., rural and non-rural or MUA versus non-MUA) being examined. Much of the work examining rural vs non-rural differences in behaviors has called for the exploration of ‘lifestyle’ factors to fully understand what rural means beyond living in a specific zip code or rural classification [61,68]. For example, within the same RUCC categorization, someone may live in or out of town (affecting drive times to employment or other access to other resources) or live and work on a farm (affecting number, timing and length of workdays) [68].

This study additionally has many strengths. The analysis leveraged the ECHO Cohort’s large, geographically diverse, population-based sample that included children and adolescents from diverse racial, ethnic, and socioeconomic backgrounds. In addition, this study was able to describe differences across various developmental groups, addressing a gap in the literature. An additional strength is that this investigation measured differences in a larger variety of sleep domains, including sleep duration, naps, latency, and sleep ecology.

## 5. Conclusions

This study is the first to evaluate differences in sleep outcomes based on rurality and MUA status in the United States. The results indicated that youth living in rural areas and MUAs varied from their counterparts in different ways. Broadly, findings suggested more consistent differences in sleep health for MUA populations (e.g., more likely to have not met sleep recommendations, later bedtimes), while minimal differences emerged between rural and non-rural groups. Future research may focus on the dissemination of established sleep interventions through accessible means (e.g., online programs) or accessible locations (e.g., community centers, schools) for those in MUAs to better support the sleep health and ecology of all children.

## Supplementary Material

Online Supplement

Appendix A

Supplement File A

## Figures and Tables

**Table 1:** Demographic characteristics of ECHO children, by age group.

Characteristics	Toddlers N (%)	Preschoolers N (%)	School-age Children N (%)	Adolescents N (%)	All Children
Number of Children	9685	10943	9108	2750	22234
**Child Sex**					
Male	4917 (50.8 %)	5641 (51.5 %)	4665 (51.2 %)	1419 (51.6 %)	11390 (51.2 %)
Female	4767 (49.2 %)	5302 (48.5 %)	4443 (48.8 %)	1331 (48.4 %)	10843 (48.8 %)
Missing	1				1
**Child Race**					
American Indian or Alaska Native	165 (1.8 %)	127 (1.2 %)	149 (1.7 %)	31 (1.1 %)	355 (1.7 %)
Asian	529 (5.7 %)	552 (5.2 %)	201 (2.2 %)	29 (1.1 %)	879 (4.1 %)
Black	1702 (18.4 %)	2011 (19 %)	1510 (16.8 %)	805 (29.7 %)	3630 (16.9 %)
Multiple Race	1030 (11.1 %)	1281 (12.1 %)	954 (10.6 %)	243 (9 %)	2259 (10.5 %)
Other Race	713 (7.7 %)	480 (4.5 %)	176 (2 %)	36 (1.3 %)	1025 (4.8 %)
Native Hawaiian or other Pacific Islander	75 (0.8 %)	25 (0.2 %)	17 (0.2 %)	5 (0.2 %)	91 (0.4 %)
White	5031 (54.4 %)	6132 (57.8 %)	5981 (66.5 %)	1561 (57.6 %)	13267 (61.7 %)
Missing	440	335	120	40	728
**Child Ethnicity**					
Hispanic	3020 (31.9 %)	2710 (24.8 %)	1369 (15 %)	274 (10 %)	5189 (23.6 %)
Non-Hispanic	6440 (68.1 %)	8199 (75.2 %)	7730 (85 %)	2475 (90 %)	16784 (76.4 %)
Missing	225	34	9	1	261
**Maternal Education**					
Less than high school	782 (10.2 %)	613 (7.5 %)	402 (7.8 %)	181 (21.1 %)	1333 (9.2 %)
High school degree, GED or equivalent	1479 (19.4 %)	1358 (16.7 %)	856 (16.5 %)	29 1(34 %)	2520 (17.4 %)
Some college, no degree, Associate degree (AA, AS), Trade school	1713 (22.4 %)	1879 (23.1 %)	1282 (24.8 %)	209 (24.4 %)	3433 (23.8 %)
Bachelor’s degree (BA, BS)	1870 (24.5 %)	2197 (27 %)	1286 (24.9 %)	114 (13.3 %)	3631 (25.1 %)
Master’s degree (MA, MS, Med, MSW, MBA, MPH), Professional or Doctorate Degree (PhD, EdD, MD, JD)	1799 (23.5 %)	2097 (25.7 %)	1348 (26.1 %)	62 (7.2 %)	3537 (24.5 %)
Missing	2042	2799	3934	1893	7780
**Total Household Income**					
<$30,000	1578 (30.7 %)	1809 (37.3 %)	1422 (46.7 %)	377 (66.7 %)	3154 (34.8 %)
$30,000-$49,999	595 (11.6 %)	587 (12.1 %)	494 (16.2 %)	103 (18.2 %)	1181 (13 %)
$50,000-$74,999	564 (11 %)	659 (13.6 %)	487 (16 %)	27 (4.8 %)	1149 (12.7 %)
$75,000-$99,999	530 (10.3 %)	399 (8.2 %)	166 (5.4 %)	<30	801 (8.8 %)
$100,000-$199,999	1193 (23.2 %)	1033 (21.3 %)	356 (11.7 %)	29 (5.1 %)	1854 (20.5 %)
$200,000 or more	682 (13.3 %)	361 (7.4 %)	121 (4 %)	<5	920 (10.2 %)
Missing	4543	6095	6062	2185	13175
**Source of sleep measurements**					
Parent report only	9685 (100 %)	10943 (100 %)	6552 (71.9 %)	597 (21.7 %)	17680 (79.5 %)
Child self-report only			1746 (19.2 %)	1466 (53.3 %)	1721 (7.7 %)
Both parent and child self-report			810 (8.9 %)	687 (25 %)	2833 (12.7 %)
**Census tract percent with no high school diploma (age 25+) at visit**					
Mean (SD)	13.9 (12)	11.7 (10.7)	8.9 (8.2)	10.2 (8.7)	11.5 (10.6)
Missing	94	76	15	11	76
**Socioeconomic status theme SVI score** ^a^					
Mean (SD)	0.5 (0.3)	0.5 (0.3)	0.4 (0.3)	0.5 (0.3)	0.4 (0.3)
Missing	96	77	15	11	78
**Medically Underserved Community** ^b^					
No	6356 (65.6 %)	7574 (69.2 %)	6666 (73.2 %)	1734 (63.1 %)	15258 (68.6 %)
Yes	3329 (34.4 %)	3369 (30.8 %)	2442 (26.8 %)	1016 (36.9 %)	6976 (31.4 %)
**Rural** ^c^					
Yes	441 (4.6 %)	888 (8.1 %)	748 (8.2 %)	375 (13.7 %)	1853 (8.4 %)
No	9200 (95.4 %)	10036 (91.9 %)	8346 (91.8 %)	2364 (86.3 %)	20321 (91.6 %)
Missing	44	19	14	11	60
**Medically Underserved Community**^b^ **and Rural**^c^	241	334	286	236	908

Age groups are defined as: toddlers (1–2 years), preschoolers (3–5 years), school-age children (6–12 years), and adolescents (13–17 years).

a Census tract-level socioeconomic status theme from the Centers for Disease Prevention and Control Social Vulnerability Index. The index ranges from 0 to 1, with higher values indicating higher socioeconomic vulnerability.

b Medically underserved areas were defined using MUA designations from the Health Resources & Services Administration (Index of Medical Underservice Score of less than or equal to 62.0 indicates a MUA, scores range from 0 to 100).

c Rural status was defined using the USDA’s Rural-Urban Continuum Codes (RUCC): a RUCC of 1–3 was considered a non-rural county, and 4–9 was considered a rural county.

**Table 2:** Parent reported sleep outcomes among toddlers (1–2 years).

Sleep Outcomes	Rural^a^	Non-Rural	Medically Underserved^b^	Not Medically Underserved	Overall
Children, n (%)	441 (4.6 %)	9200 (95.4 %)	3329 (34.4 %)	6356 (65.6 %)	9685
Average total nighttime sleep, hours					
Mean (SD)	11 (1.5)	10.9 (1.3)	**10.8 (1.5)**	**11 (1.2)**	10.9 (1.3)
Missing, n	377	6586	2632	4362	6994
Average total 24-h sleep, hours					
Mean (SD)	12.6 (1.3)	12.7 (1.8)	**12.3 (1.9)**	**12.9 (1.7)**	12.7 (1.8)
Missing, n	390	7873	2780	5522	8302
Meeting sleep recommendations for 24-h sleep^c^, n (%)					
Yes	<50	1159 (87.3 %)	**450 (82.0 %)**	**762 (91.4 %)**	1212 (87.6 %)
No	<5	168 (12.7 %)	**99 (18.0 %)**	**72 (8.6 %)**	171 (12.4 %)
Missing	390	7873	2780	5522	
Wake time on weekdays, hh:mm					
Mean (SD)	**06:58 (00:56)**	**07:22 (01:10)**	**07:29 (01:22)**	**07:18 (01:04)**	07:21 (01:09)
Missing, n	210	5892	2406	3725	6131
Wake time on weekend, hh:mm					
Mean (SD)	**07:19 (01:04)**	**07:43 (01:16)**	**07:53 (01:29)**	**07:37 (01:10)**	07:41 (01:16)
Missing, n	216	5961	2435	3771	6206
Bedtime on weekdays, hh:mm					
Mean (SD)	**20:05 (00:55)**	**20:37 (01:10)**	**20:56 (01:15)**	**20:26 (01:05)**	20:35 (01:09)
Missing, n	201	5644	2256	3618	5874
Bedtime on weekend, hh:mm					
Mean (SD)	**20:19 (01:02)**	**20:50 (01:16)**	**21:10 (01:19)**	**20:40 (01:12)**	20:48 (01:15)
Missing, n	211	5720	2289	3671	5960
Time to fall asleep, minutes					
Mean (SD)	**18.6 (15)**	**22.6 (19.4)**	**25.9 (24.5)**	**21.3 (17.4)**	22.4 (19.4)
Missing, n	222	6367	2600	4029	6629
Frequency of delayed bedtime, n (%)					
Never or Almost Never	253 (61.6 %)	3290 (56.7 %)	1161 (57.9 %)	2398 (56.7 %)	3559 (57.1 %)
Sometimes	124 (30.2 %)	1944 (33.5 %)	668 (33.3 %)	1404 (33.2 %)	2072 (33.2 %)
Almost Always or Always	34 (8.3 %)	570 (9.8 %)	175 (8.7 %)	429 (10.1 %)	604 (9.7 %)
Missing	30	3396	1325	2125	3450
Total naptime, minutes					
Mean (SD)	**122.1 (37.3)**	**115 (49.1)**	**122.1 (55.2)**	**112.4 (45.4)**	115.3 (48.7)
Missing, n	207	3620	1582	2272	3854
Naps per week, n (%)					
1 or fewer days/week	18 (7.9 %)	165 (9.8 %)	**78 (12.5 %)**	**105 (8.1 %)**	183 (9.6 %)
2–3 days/week	12 (5.3 %)	130 (7.7 %)	**54 (8.7 %)**	**89 (6.9 %)**	143 (7.5 %)
4–5 days/week	16 (7 %)	167 (9.9 %)	**80 (12.8 %)**	**103 (8 %)**	183 (9.6 %)
6–7 days/week	181 (79.7 %)	1220 (72.5 %)	**412 (66 %)**	**992 (77 %)**	1404(73.4 %)
Missing	214	7518	2705	5067	7772

**Bolded** values indicate p-value of <0.05. To compare rural to non-rural and medically underserved to not medically underserved, respectively, we used t-tests for continuous variables and chi-squared tests for categorical variables.

a Rural status was defined using the USDA’s Rural-Urban Continuum Codes (RUCC): a RUCC of 1–3 was considered a non-rural county, and 4–9 was considered a rural county.

b Medically underserved areas were defined using MUA designations from the Health Resources & Services Administration (Index of Medical Underservice Score of less than or equal to 62.0 indicates a MUA, scores range from 0 to 100).

c Reporting at least 11 h of sleep per 24 h.

**Table 3:** Parent reported sleep outcomes among preschoolers (3–5 years).

Sleep Outcomes	Rural^a^	Non-Rural	Medically Underserved^b^	Not Medically Underserved	Overall
Children, n (%)	888 (8.1 %)	10036 (91.9 %)	3369 (30.8 %)	7574 (69.2 %)	10943
Average total nighttime sleep, hours					
Mean (SD)	10.9 (1.4)	10.9 (1.4)	10.9 (1.5)	10.9 (1.3)	10.9 (1.4)
Missing, n	633	5985	2456	4174	6630
Average total 24-h sleep, hours					
Mean (SD)	**11.3 (1.6)**	**11.7 (1.4)**	11.7 (1.7)	11.7 (1.3)	11.7 (1.4)
Missing, n	679	7564	2740	5521	8261
Meeting sleep recommendations for 24-h sleep^c^, n (%)					
Yes	**179 (85.6 %)**	**2337 (94.5 %)**	**577 (91.7 %)**	**1940 (94.5 %)**	2517 (93.8 %)
No	**30 (14.4 %)**	**135 (5.5 %)**	**52 (8.3 %)**	**113 (5.5 %)**	165 (6.2 %)
Missing	679	7564	2740	5521	8261
Wake time on weekdays, hh:mm					
Mean (SD)	**06:52 (00:52)**	**07:21 (01:04)**	**07:26 (01:15)**	**07:14 (00:59)**	07:17 (01:03)
Missing, n	220	5332	2117	3446	5563
Wake time on weekend, hh:mm					
Mean (SD)	**07:03 (00:58)**	**07:55 (01:16)**	**08:06 (01:28)**	**07:43 (01:11)**	07:48 (01:16)
Missing, n	224	5538	2174	3599	5773
Bedtime on weekdays, hh:mm					
Mean (SD)	**20:09 (00:50)**	**20:44 (00:58)**	**20:51 (01:02)**	**20:35 (00:56)**	20:40 (00:58)
Missing, n	197	4827	1815	3220	5035
Bedtime on weekend, hh:mm					
Mean (SD)	**20:18 (00:55)**	**21:10 (01:10)**	**21:18 (01:16)**	**20:59 (01:07)**	21:04 (01:10)
Missing, n	210	5029	1879	3371	5250
Time to fall asleep, minutes					
Mean (SD)	26.7 (22.9)	26 (22.4)	**29.4 (27)**	**25.1 (20.8)**	26.1 (22.4)
Missing, n	586	5576	2289	3885	6174
Frequency of delayed bedtime, n (%)					
Never or Almost Never	231 (49.5 %)	4385 (49.2 %)	**1573 (53.8 %)**	**3054 (47.2 %)**	4627 (49.2 %)
Sometimes	168 (36 %)	3150 (35.3 %)	**1013 (34.6 %)**	**2311 (35.7 %)**	3324 (35.4 %)
Almost Always or Always	68 (14.6 %)	1377 (15.5 %)	**340 (11.6 %)**	**1107 (17.1 %)**	1447 (15.4 %)
Missing	421	1124	443	1102	1545
Total naptime, minutes					
Mean (SD)	90 (38.8)	91.7 (45.4)	**96.4 (49.5)**	**89.9 (43.4)**	91.6 (45.2)
Missing, n	686	6233	2311	4625	6936
Naps per week, n (%)					
1 or fewer days/week	**177 (52.5 %)**	**2175 (44 %)**	**475 (37.6 %)**	**1885 (46.8 %)**	2360 (44.6 %)
2–3 days/week	**71 (21.1 %)**	**944 (19.1 %)**	**278 (22 %)**	**737 (18.3 %)**	1015 (19.2 %)
4–5 days/week	**53 (15.7 %)**	**962 (19.5 %)**	**262 (20.7 %)**	**753 (18.7 %)**	1015 (19.2 %)
6–7 days/week	**36 (10.7 %)**	**862 (17.4 %)**	**249 (19.7 %)**	**650 (16.1 %)**	899 (17 %)
Missing	551	5093	2105	3549	5654

**Bolded** values indicate p-value of <0.05. To compare rural to non-rural and medically underserved to not medically underserved, respectively, we used t-tests for continuous variables and chi-squared tests for categorical variables.

a Rural status was defined using the USDA’s Rural-Urban Continuum Codes (RUCC): a RUCC of 1–3 was considered a non-rural county, and 4–9 was considered a rural county.

b Medically underserved areas were defined using MUA designations from the Health Resources & Services Administration (Index of Medical Underservice Score of less than or equal to 62.0 indicates a MUA, scores range from 0 to 100).

c Reporting at least 10 h of sleep per 24 h.

**Table 4:** Parent reported sleep outcomes among school-age children (6–12 years).

Sleep Outcomes	Rural^a^	Non-Rural	Medically Underserved^b^	Not Medically Underserved	Overall
Children, n (%)	567 (7.7 %)	6781 (92.3 %)	2093 (28.4 %)	5269 (71.6 %)	7362
Average total nighttime sleep, hours					
Mean (SD)	10.3 (1.3)	10.5 (1.4)	10.4 (1.5)	10.5 (1.4)	10.4 (1.4)
Missing, n	325	2195	710	1813	2523
Meeting sleep recommendations^c^, n (%)					
Yes	235 (97.1 %)	4325 (94.3 %)	**1275 (92.2 %)**	**3296 (95.4 %)**	4571 (94.5 %)
No	7 (2.9 %)	261 (5.7 %)	**108 (7.8 %)**	**160 (4.6 %)**	268 (5.5 %)
Missing	325	2195	710	1813	2523
Wake time on weekdays, hh:mm					
Mean (SD)	**06:46 (00:44)**	**07:01 (00:55)**	07:02 (01:04)	06:59 (00:50)	07:00 (00:54)
Missing, n	51	714	216	549	765
Wake time on weekend, hh:mm					
Mean (SD)	**07:38 (01:08)**	**08:06 (01:22)**	**08:21 (01:33)**	**07:56 (01:14)**	08:04 (01:21)
Missing, n	64	871	260	675	935
Bedtime on weekdays, hh:mm					
Mean (SD)	**20:32 (00:43)**	**20:50 (00:49)**	**20:53 (00:52)**	**20:46 (00:47)**	20:48 (00:48)
Missing, n	39	620	187	473	660
Bedtime on weekend, hh:mm					
Mean (SD)	**21:15 (01:04)**	**21:42 (01:10)**	**21:54 (01:16)**	**21:34 (01:06)**	21:39 (01:10)
Missing, n	48	779	227	601	828
Time to fall asleep, minutes					
Mean (SD)	**25.2 (21.3)**	**27.1 (24.3)**	**29 (25.9)**	**26.1 (23.3)**	26.9 (24.1)
Missing, n	118	1482	436	1164	1600
Frequency of delayed bedtime, n (%)					
Never or Almost Never	232 (48.3 %)	2690 (45.3 %)	**926 (47.8 %)**	**2005 (44.6 %)**	2931 (45.5 %)
Sometimes	165 (34.4 %)	2169 (36.5 %)	**706 (36.5 %)**	**1631 (36.2 %)**	2337 (36.3 %)
Almost Always or Always	83 (17.3 %)	1083 (18.2 %)	**304 (15.7 %)**	**864 (19.2 %)**	1168 (18.1 %)
Missing	87	839	157	769	926
Total naptime, minutes					
Mean (SD)	**82.8 (41.3)**	**94.6 (50.9)**	**100.6 (51.2)**	**89.2 (49.5)**	93.8 (50.5)
Missing, n	507	5630	1600	4547	6147
Naps per week, n (%)					
1 or fewer days/week	**442 (93.2 %)**	**5007 (88.5 %)**	**1494 (83.1 %)**	**3968 (91.3 %)**	5462 (88.9 %)
2–3 days/week	**15 (3.2 %)**	**455 (8 %)**	**207 (11.5 %)**	**264 (6.1 %)**	471 (7.7 %)
4–5 days/week	**8 (1.7 %)**	**96 (1.7 %)**	**45 (2.5 %)**	**59 (1.4 %)**	104 (1.7 %)
6–7 days/week	**9 (1.9 %)**	**98 (1.7 %)**	**51 (2.8 %)**	**56 (1.3 %)**	107 (1.7 %)
Missing	93	1125	296	922	1218

**Bolded** values indicate p-value of <0.05. To compare rural to non-rural and medically underserved to not medically underserved, respectively, we used t-tests for continuous variables and chi-squared tests for categorical variables.

a Rural status was defined using the USDA’s Rural-Urban Continuum Codes (RUCC): a RUCC of 1–3 was considered a non-rural county, and 4–9 was considered a rural county.

b Medically underserved areas were defined using MUA designations from the Health Resources & Services Administration (Index of Medical Underservice Score of less than or equal to 62.0 indicates a MUA, scores range from 0 to 100).

c Reporting at least 9 h of nighttime sleep.

**Table 5:** Self-reported sleep outcomes among adolescents (13–17 years).

Sleep Outcomes	Rural^a^	Non-Rural	Medically Underserved^b^	Not Medically Underserved	Overall
Children, n (%)	317 (14.8 %)	1825 (85.2 %)	825 (38.3 %)	1328 (61.7 %)	2153
Average total nighttime sleep, hours					
Mean (SD)	9.7 (2.4)	9.5 (2.5)	**9.8 (2.7)**	**9.5 (2.3)**	9.6 (2.5)
Missing, n	18	202	71	149	220
Meeting sleep recommendations^c^, n (%)					
Yes	241 (80.6 %)	1283 (79.1 %)	596(79 %)	936(79.4 %)	1532 (79.3 %)
No	58 (19.4 %)	340 (20.9 %)	158(21 %)	243(20.6 %)	401 (20.7 %)
Missing	18	202	71	149	220
Wake time on weekdays, hh:mm					
Mean (SD)	07:39 (01:47)	07:29 (01:45)	**07:45 (01:53)**	**07:22 (01:40)**	07:30 (01:45)
Missing, n	14	174	78	110	188
Wake time on weekend, hh:mm					
Mean (SD)	09:38 (02:00)	09:45 (01:50)	**09:59 (02:00)**	**09:35 (01:46)**	09:44 (01:52)
Missing, n	27	219	102	146	248
Bedtime on weekdays, hh:mm					
Mean (SD)	22:30 (01:22)	22:39 (01:17)	**22:44 (01:26)**	**22:34 (01:13)**	22:38 (01:18)
Missing, n	32	244	114	165	279
Bedtime on weekend, hh:mm					
Mean (SD)	**23:38 (01:38)**	**23:59 (01:27)**	**00:05 (01:34)**	**23:51 (01:26)**	23:56 (01:29)
Missing, n	34	288	142	182	324
Time to fall asleep, minutes					
Mean (SD)	38.9 (37.3)	36 (35.4)	**39.2 (38.9)**	**34.8 (33.6)**	36.5 (35.8)
Missing, n	21	162	68	115	183
Frequency of delayed bedtime, n (%)					
Never or Almost Never	96 (30.5 %)	566 (31.9 %)	253(31 %)	414(32.2 %)	667 (31.7 %)
Sometimes	129(41 %)	725 (40.8 %)	340(41.7 %)	516(40.1 %)	856 (40.7 %)
Almost Always or Always	90 (28.6 %)	485 (27.3 %)	223(27.3 %)	356(27.7 %)	579 (27.5 %)
Missing	2	49	9	42	51
Total naptime, minutes					
Mean (SD)	106.4 (54.6)	110.6 (59.9)	**118.2 (60.7)**	**103.9 (57.2)**	110.3 (59.2)
Missing, n	166	1004	389	786	1175
Naps per week, n (%)					
1 or fewer days/week	222 (70.5 %)	1200 (67.3 %)	**506(61.9 %)**	**925(71.6 %)**	1431 (67.8 %)
2–3 days/week	58 (18.4 %)	385 (21.6 %)	**200(24.4 %)**	**245(19 %)**	445 (21.1 %)
4–5 days/week	23(7.3 %)	119 (6.7 %)	**63(7.7 %)**	**79(6.1 %)**	142 (6.7 %)
6–7 days/week	12(3.8 %)	80(4.5 %)	**49(6 %)**	**43(3.3 %)**	92(4.4 %)
Missing	2	41	7	36	43

**Bolded** values indicate p-value of <0.05. To compare rural to non-rural and medically underserved to not medically underserved, respectively, we used t-tests for continuous variables and chi-squared tests for categorical variables.

a Rural status was defined using the USDA’s Rural-Urban Continuum Codes (RUCC): a RUCC of 1–3 was considered a non-rural county, and 4–9 was considered a rural county.

b Medically underserved areas were defined using MUA designations from the Health Resources & Services Administration (Index of Medical Underservice Score of less than or equal to 62.0 indicates a MUA, scores range from 0 to 100).

c Reporting at least 8 h of nighttime sleep.

## Data Availability

Select de-identified data from the ECHO Program are available through NICHD’s Data and Specimen Hub (DASH). Information on study data not available on DASH, such as some Indigenous datasets, can be found on the ECHO study DASH webpage.

## Appendix A. Supplementary data

Supplementary data to this article can be found online at https://doi.org/10.1016/j.sleep.2025.108754.
