# Supplementary material for: Exploring sleep outcomes in youth across settings: Are there differences based on rurality or medically underserved status in the ECHO cohort?
Source: Sleep Med. Author manuscript; Available in PMC 2026 Apr 1. (PMC13043269; doi:10.1016/j.sleep.2025.108754)
Supplement: Online Supplement [file NIHMS2157873-supplement-Online_Supplement.docx]

Supplement Table B. Self-Report Sleep Outcomes, School-age Children

| **Sleep Outcomes** | **Rural^1^** | **Non-Rural** | **Medically Underserved^2^** | **Not Medically Underserved** | **Overall** |
| --- | --- | --- | --- | --- | --- |
| Children, N | 247 | 2304 | 543 | 2013 | 2556 |
| Average total nighttime sleep, hours |  |  |  |  |  |
| Mean (SD) | 10.1 (1.4) | 10.2 (1.5) | 10.2 (1.6) | 10.2 (1.5) | 10.2 (1.5) |
| Missing, n | 38 | 215 | 50 | 203 | 253 |
| Wake time on weekdays, hh:mm |  |  |  |  |  |
| Mean (SD) | **06:36 (00:49)** | **07:00 (01:04)** | 06:54 (01:05) | 06:59 (01:03) | 06:58 (01:03) |
| Missing, n | 18 | 122 | 36 | 104 | 140 |
| Wake time on weekend, hh:mm |  |  |  |  |  |
| Mean (SD) | **07:47 (01:10)** | **08:07 (01:28)** | 08:10 (01:33) | 08:04 (01:25) | 08:05 (01:27) |
| Missing, n | 25 | 188 | 39 | 174 | 213 |
| Bedtime on weekdays, hh:mm |  |  |  |  |  |
| Mean (SD) | 20:50 (00:53) | 20:56 (00:57) | 20:57 (00:58) | 20:55 (00:56) | 20:56 (00:57) |
| Missing, n | 15 | 94 | 21 | 88 | 109 |
| Bedtime on weekend, hh:mm |  |  |  |  |  |
| Mean (SD) | **21:42 (01:09)** | **21:52 (01:16)** | 21:56 (01:13) | 21:50 (01:16) | 21:51 (01:15) |
| Missing, n | 21 | 145 | 35 | 131 | 166 |
| Time to fall asleep, minutes |  |  |  |  |  |
| Mean (SD) | 29.4 (27.2) | 29.5 (28.8) | 30.5 (31.6) | 29.2 (27.7) | 29.5 (28.6) |
| Missing, n | 10 | 133 | 30 | 113 | 143 |
| Frequency of delayed bedtime, n (%) |  |  |  |  |  |
| Never or Almost Never | 101(42.3%) | 1007(45.1%) | **274(51.5%)** | **836(43%)** | 1110(44.8%) |
| Sometimes | 98(41%) | 848(38%) | **182(34.2%)** | **767(39.5%)** | 949(38.3%) |
| Almost Always or Always | 40(16.7%) | 377(16.9%) | **76(14.3%)** | **341(17.5%)** | 417(16.8%) |
| Missing | 8 | 72 | 11 | 69 | 80 |
| Total naptime, minutes |  |  |  |  |  |
| Mean (SD) | 78.3 (49.5) | 85.8 (58.6) | 91.1 (60.7) | 82.9 (56.8) | 85.1 (57.9) |
| Missing, n | 207 | 1847 | 409 | 1649 | 2058 |
| Naps per week, n (%) |  |  |  |  |  |
| 1 or fewer days/week | 226(92.6%) | 2003(89.9%) | **452(85.3%)** | **1782(91.5%)** | 2234(90.2%) |
| 2-3 days/week | 17(7%) | 147(6.6%) | **52(9.8%)** | **112(5.7%)** | 164(6.6%) |
| 4-5 days/week | <5 | <40 | **11(2.1%)** | **29(1.5%)** | 40(1.6%) |
| 6-7 days/week |  | 40(1.8%) | **15(2.8%)** | **25(1.3%)** | 40(1.6%) |
| Missing | <5 | <80 | 13 | 65 | 78 |

**Bolded** values indicate p-value of < 0.05. To compare rural to non-rural and medically underserved to not medically underserved, respectively, we used t-tests for continuous variables and chi-squared tests for categorical variables.

1 Rural status was defined using the USDA’s Rural-Urban Continuum Codes (RUCC): a RUCC of 1-3 was considered a non-rural county, and 4-9 was considered a rural county.

2 Medically underserved areas were defined using MUA designations from the Health Resources & Services Administration (Index of Medical Underservice Score of less than or equal to 62.0 indicates a MUA, scores range from 0 to 100).

3 Reporting at least 9 hours of nighttime sleep.

Supplement Table C. Parent-Report Sleep Outcomes, Adolescents

| **Sleep Outcomes** | **Rural^1^** | **Non-Rural** | **Medically Underserved^2^** | **Not Medically Underserved** | **Overall** |
| --- | --- | --- | --- | --- | --- |
| Children, N | 257 | 1027 | 553 | 731 | 1284 |
| Average total nighttime sleep, hours |  |  |  |  |  |
| Mean (SD) | 9.9 (2) | 9.7 (1.9) | 9.9 (1.9) | 9.7 (1.9) | 9.8 (1.9) |
| Missing, n | 149 | 619 | 305 | 463 | 768 |
| Wake time on weekdays, hh:mm |  |  |  |  |  |
| Mean (SD) | **07:21 (01:22)** | **07:08 (01:17)** | 07:15 (01:26) | 07:07 (01:12) | 07:10 (01:18) |
| Missing, n | 50 | 176 | 106 | 120 | 226 |
| Wake time on weekend, hh:mm |  |  |  |  |  |
| Mean (SD) | 09:32 (01:33) | 09:31 (01:36) | **09:48 (01:37)** | **09:19 (01:33)** | 09:31 (01:35) |
| Missing, n | 73 | 230 | 150 | 153 | 303 |
| Bedtime on weekdays, hh:mm |  |  |  |  |  |
| Mean (SD) | 22:04 (00:55) | 22:02 (01:03) | 21:59 (01:01) | 22:05 (01:01) | 22:02 (01:01) |
| Missing, n | 62 | 194 | 123 | 133 | 256 |
| Bedtime on weekend, hh:mm |  |  |  |  |  |
| Mean (SD) | 23:24 (01:10) | 23:15 (01:13) | 23:22 (01:15) | 23:13 (01:11) | 23:17 (01:12) |
| Missing, n | 89 | 259 | 185 | 163 | 348 |
| Time to fall asleep, minutes |  |  |  |  |  |
| Mean (SD) | 32.5 (27.8) | 34.6 (30.4) | **37.7 (32)** | **31.9 (28.2)** | 34.2 (29.9) |
| Missing, n | 98 | 316 | 206 | 208 | 414 |
| Frequency of delayed bedtime, n (%) |  |  |  |  |  |
| Never or Almost Never | 101(41.7%) | 403(44.5%) | **214(41.8%)** | **290(45.7%)** | 504(43.9%) |
| Sometimes | 97(40.1%) | 335(37%) | **218(42.6%)** | **214(33.7%)** | 432(37.7%) |
| Almost Always or Always | 44(18.2%) | 167(18.5%) | **80(15.6%)** | **131(20.6%)** | 211(18.4%) |
| Missing | 15 | 122 | 41 | 96 | 137 |
| Total naptime, minutes |  |  |  |  |  |
| Mean (SD) | 111.1 (41.4) | 106.8 (46.1) | **113.2 (44.8)** | **101.3 (44.1)** | 107.9 (44.9) |
| Missing, n | 148 | 719 | 320 | 547 | 867 |
| Naps per week, n (%) |  |  |  |  |  |
| 1 or fewer days/week | **138(57.7%)** | **671(74.4%)** | **297(58.6%)** | **512(80.8%)** | 809(70.9%) |
| 2-3 days/week | **62(25.9%)** | **130(14.4%)** | **124(24.5%)** | **68(10.7%)** | 192(16.8%) |
| 4-5 days/week | **20(8.4%)** | **53(5.9%)** | **45(8.9%)** | **28(4.4%)** | 73(6.4%) |
| 6-7 days/week | **19(7.9%)** | **48(5.3%)** | **41(8.1%)** | **26(4.1%)** | 67(5.9%) |
| Missing | 18 | 125 | 46 | 97 | 143 |

**Bolded** values indicate p-value of < 0.05. To compare rural to non-rural and medically underserved to not medically underserved, respectively, we used t-tests for continuous variables and chi-squared tests for categorical variables.

1 Rural status was defined using the USDA’s Rural-Urban Continuum Codes (RUCC): a RUCC of 1-3 was considered a non-rural county, and 4-9 was considered a rural county.

2 Medically underserved areas were defined using MUA designations from the Health Resources & Services Administration (Index of Medical Underservice Score of less than or equal to 62.0 indicates a MUA, scores range from 0 to 100).

Supplement Table D. Parent-Reported Sleep Ecology Outcomes, Toddlers

| **Sleep Ecology Outcomes in the past 7 days** | **Medically Underserved^1^ Are** | **Not a Medically Underserved Are** | **p-value** | **Overall** |
| --- | --- | --- | --- | --- |
| Total number of children, N | 33 | 89 |  | 122 |
| ***Played video or computer games just before falling asleep, n (%)*** |  |  | 0.0661 |  |
| Never | 24(72.7%) | 80(89.9%) |  | 104(85.2%) |
| Almost Never | <5 | <5 |  | 8(6.6%) |
| Sometimes | <10 | <5 |  | <10 |
| Almost Always |  | <5 |  | <5 |
| Always |  | <5 |  | <5 |
| Missing |  |  |  |  |
| ***Watched TV shows or videos just before falling asleep, n (%)*** |  |  | 0.7809 |  |
| Never | 9(28.1%) | 33(37.1%) |  | 42(34.7%) |
| Almost Never | 6(18.8%) | 10(11.2%) |  | 16(13.2%) |
| Sometimes | 11(34.4%) | 27(30.3%) |  | 38(31.4%) |
| Almost Always | <5 | <15 |  | 17(14%) |
| Always | <5 | <10 |  | 8(6.6%) |
| Missing | 1 |  |  | 1 |
| ***Used a phone, computer, or electronic device just before falling asleep, n (%)*** |  |  | 0.0999 |  |
| Never | 18(54.5%) | 67(75.3%) |  | 85(69.7%) |
| Almost Never | 6(18.2%) | 9(10.1%) |  | 15(12.3%) |
| Sometimes | <10 | <10 |  | 16(13.1%) |
| Almost Always | <5 | <5 |  | <5 |
| Always |  | <5 |  | <5 |
| Missing |  |  |  |  |
| ***Needed someone with him/her to fall asleep, n (%)*** |  |  | 0.5035 |  |
| Never | 15(45.5%) | 43(48.3%) |  | 58(47.5%) |
| Almost Never | <5 | <20 |  | 19(15.6%) |
| Sometimes | 6(18.2%) | 10(11.2%) |  | 16(13.1%) |
| Almost Always | 5(15.2%) | 7(7.9%) |  | 12(9.8%) |
| Always | <5 | <15 |  | 17(13.9%) |
| Missing |  |  |  |  |
| ***Followed a bedtime routine before falling asleep, n (%)*** |  |  | 0.0249 |  |
| Never | <5 | <5 |  | <5 |
| Almost Never | <5 | <5 |  | <10 |
| Sometimes | 9(27.3%) | 12(13.5%) |  | 21(17.2%) |
| Almost Always | 11(33.3%) | 31(34.8%) |  | 42(34.4%) |
| Always | 8(24.2%) | 43(48.3%) |  | 51(41.8%) |
| Missing |  |  |  |  |
| ***Woke up at about the same time every day, n (%)*** |  |  | 0.0989 |  |
| Never |  | <5 |  | <5 |
| Almost Never | <5 | <5 |  | <10 |
| Sometimes | <15 | <15 |  | 24(19.7%) |
| Almost Always | 14(42.4%) | 42(47.2%) |  | 56(45.9%) |
| Always | 6(18.2%) | 30(33.7%) |  | 36(29.5%) |
| Missing |  |  |  |  |
| ***Tried to fall asleep at about the same time every day, n (%)*** |  |  | 0.0237 |  |
| Never | <5 | <5 |  | <5 |
| Almost Never | <5 | <5 |  | <10 |
| Sometimes | 10(30.3%) | 10(11.2%) |  | 20(16.4%) |
| Almost Always | 13(39.4%) | 45(50.6%) |  | 58(47.5%) |
| Always | 5(15.2%) | 29(32.6%) |  | 34(27.9%) |
| Missing |  |  |  |  |

1 Medically underserved areas were defined using MUA designations from the Health Resources & Services Administration (Index of Medical Underservice Score of less than or equal to 62.0 indicates a MUA, scores range from 0 to 100).

Supplement Table E. Parent-Reported Sleep Ecology Outcomes, Preschoolers

| **Sleep Ecology Outcomes in the past 7 days** | **Medically Underserved^1^** | **Not a Medically Underserved** | **p-value** | **Overall** |
| --- | --- | --- | --- | --- |
| Total number of children | 68 | 269 |  | 337 |
| ***Played video or computer games just before falling asleep, n (%)*** |  |  | 0.0016 |  |
| Never | 38(55.9%) | 204(75.8%) |  | 242(71.8%) |
| Almost Never | 8(11.8%) | 30(11.2%) |  | 38(11.3%) |
| Sometimes | <20 | <30 |  | 48(14.2%) |
| Almost Always | <5 | <10 |  | 9(2.7%) |
| Always |  |  |  |  |
| Missing |  |  |  |  |
| ***Watched TV shows or videos just before falling asleep, n(%)*** |  |  | 0.0192 |  |
| Never | 11(16.2%) | 64(23.8%) |  | 75(22.3%) |
| Almost Never | 8(11.8%) | 61(22.7%) |  | 69(20.5%) |
| Sometimes | 24(35.3%) | 91(33.8%) |  | 115(34.1%) |
| Almost Always | <25 | <45 |  | 63(18.7%) |
| Always | <5 | <15 |  | 15(4.5%) |
| Missing |  |  |  |  |
| ***Used a phone, computer, or electronic device just before falling asleep, n(%)*** |  |  | 0.1005 |  |
| Never | 33(49.3%) | 144(53.7%) |  | 177(52.8%) |
| Almost Never | 6(9%) | 50(18.7%) |  | 56(16.7%) |
| Sometimes | 19(28.4%) | 53(19.8%) |  | 72(21.5%) |
| Almost Always | <10 | <20 |  | 24(7.2%) |
| Always | <5 | <10 |  | 6(1.8%) |
| Missing | 1 | 1 |  | 2 |
| ***Needed someone with him/her to fall asleep, n (%)*** |  |  | 0.06360 |  |
| Never | 22(32.4%) | 114(42.4%) |  | 136(40.4%) |
| Almost Never | 14(20.6%) | 47(17.5%) |  | 61(18.1%) |
| Sometimes | 12(17.6%) | 41(15.2%) |  | 53(15.7%) |
| Almost Always | 14(20.6%) | 26(9.7%) |  | 40(11.9%) |
| Always | 6(8.8%) | 41(15.2%) |  | 47(13.9%) |
| Missing |  |  |  |  |
| ***Followed a bedtime routine before falling asleep, n (%)*** |  |  | 0.3419 |  |
| Never | <5 | <5 |  | <5 |
| Almost Never | <5 | <10 |  | <15 |
| Sometimes | 14(20.6%) | 32(11.9%) |  | 46(13.7%) |
| Almost Always | 22(32.4%) | 93(34.7%) |  | 115(34.2%) |
| Always | 28(41.2%) | 133(49.6%) |  | 161(47.9%) |
| Missing |  | 1 |  | 1 |
| ***Woke up at about the same time every day, n (%)*** |  |  | 0.6884 |  |
| Never | <5 | <5 |  | <5 |
| Almost Never | <5 | <10 |  | <15 |
| Sometimes | 14(20.6%) | 32(11.9%) |  | 46(13.7%) |
| Almost Always | 22(32.4%) | 93(34.7%) |  | 115(34.2%) |
| Always | 28(41.2%) | 133(49.6%) |  | 161(47.9%) |
| Missing | 1 | 1 |  | 2 |
| ***Tried to fall asleep at about the same time every day, n (%)*** |  |  | 0.7524 |  |
| Never |  | <5 |  | <5 |
| Almost Never |  | <5 |  | <5 |
| Sometimes | 10(14.7%) | 32(11.9%) |  | 42(12.5%) |
| Almost Always | 41(60.3%) | 152(56.7%) |  | 193(57.4%) |
| Always | 17(25%) | <85 |  | <100 |
| Missing |  | 1 |  | 1 |

1 Medically underserved areas were defined using MUA designations from the Health Resources & Services Administration (Index of Medical Underservice Score of less than or equal to 62.0 indicates a MUA, scores range from 0 to 100).

Supplement Table F. Parent-Reported Sleep Ecology Outcomes, School-age Children

| **Sleep Ecology Outcomes in the past 7 days** | **Medically Underserved^1^** | **Not a Medically Underserved** | **p-value** | **Overall** |
| --- | --- | --- | --- | --- |
| Total number of children | 49 | 269 |  | 318 |
| ***Played video or computer games just before falling asleep, n (%)*** |  |  | 0.7873 |  |
| Never | 23(47.9%) | 140(52%) |  | 163(51.4%) |
| Almost Never | 10(20.8%) | 55(20.4%) |  | 65(20.5%) |
| Sometimes | 10(20.8%) | 55(20.4%) |  | 65(20.5%) |
| Almost Always | <5 | <20 |  | 18(5.7%) |
| Always | <5 | <5 |  | 6(1.9%) |
| Missing | 1 |  |  | 1 |
| ***Watched TV shows or videos just before falling asleep, n(%)*** |  |  | 0.0853 |  |
| Never | 13(26.5%) | 60(22.3%) |  | 73(23%) |
| Almost Never | <5 | <45 |  | 48(15.1%) |
| Sometimes | 21(42.9%) | 98(36.4%) |  | 119(37.4%) |
| Almost Always | <5 | <50 |  | 53(16.7%) |
| Always | 7(14.3%) | 18(6.7%) |  | 25(7.9%) |
| Missing |  |  |  |  |
| ***Used a phone, computer, or electronic device just before falling asleep, n (%)*** |  |  | 0.5173 |  |
| Never | 18(36.7%) | 120(44.8%) |  | 138(43.5%) |
| Almost Never | 12(24.5%) | 44(16.4%) |  | 56(17.7%) |
| Sometimes | 11(22.4%) | 60(22.4%) |  | 71(22.4%) |
| Almost Always | <10 | <40 |  | 40(12.6%) |
| Always | <5 | <10 |  | 12(3.8%) |
| Missing |  | 1 |  | 1 |
| ***Needed someone with him/her to fall asleep, n (%)*** |  |  | 0.8990 |  |
| Never | 30(61.2%) | 152(57.4%) |  | 182(58%) |
| Almost Never | 5(10.2%) | 41(15.5%) |  | 46(14.6%) |
| Sometimes | 6(12.2%) | 27(10.2%) |  | 33(10.5%) |
| Almost Always | <5 | <25 |  | 27(8.6%) |
| Always | <5 | <25 |  | 26(8.3%) |
| Missing |  | 4 |  | 4 |
| ***Followed a bedtime routine before falling asleep, n (%)*** |  |  | 0.8892 |  |
| Never | <5 | <5 |  | 5(1.6%) |
| Almost Never | <5 | <15 |  | 14(4.4%) |
| Sometimes | 9(18.4%) | 36(13.4%) |  | 45(14.2%) |
| Almost Always | 21(42.9%) | 115(42.8%) |  | 136(42.8%) |
| Always | 16(32.7%) | 102(37.9%) |  | 118(37.1%) |
| Missing |  |  |  |  |
| ***Woke up at about the same time every day, n (%)*** |  |  | 0.4570 |  |
| Never | <5 | <5 |  | 5(1.6%) |
| Almost Never | <5 | <15 |  | 14(4.4%) |
| Sometimes | 9(18.4%) | 36(13.4%) |  | 45(14.2%) |
| Almost Always | 21(42.9%) | 115(42.8%) |  | 136(42.8%) |
| Always | 16(32.7%) | 102(37.9%) |  | 118(37.1%) |
| Missing |  |  |  |  |
| ***Tried to fall asleep at about the same time every day, n (%)*** |  |  | 0.3301 |  |
| Never | <5 | <10 |  | 6(1.9%) |
| Almost Never | <5 | <15 |  | 13(4.1%) |
| Sometimes | 12(24.5%) | 39(14.5%) |  | 51(16%) |
| Almost Always | 21(42.9%) | 155(57.6%) |  | 176(55.3%) |
| Always | 13(26.5%) | 59(21.9%) |  | 72(22.6%) |

1 Medically underserved areas were defined using MUA designations from the Health Resources & Services Administration (Index of Medical Underservice Score of less than or equal to 62.0 indicates a MUA, scores range from 0 to 100).

Supplement Table G. Self-Reported Sleep Ecology Outcomes, Adolescents

| **Sleep Ecology Outcomes in the past 7 days** | **Medically Underserved^1^** | **Not a Medically Underserved** | **p-value** | **Overall** |
| --- | --- | --- | --- | --- |
| Total number of children | 367 | 535 |  | 902 |
| ***Played video or computer games just before falling asleep, n (%)*** |  |  | 0.8860 |  |
| Never | 135(36.8%) | 204(38.3%) |  | 339(37.7%) |
| Almost Never | 42(11.4%) | 70(13.1%) |  | 112(12.4%) |
| Sometimes | 98(26.7%) | 130(24.4%) |  | 228(25.3%) |
| Almost Always | <50 | 66(12.4%) |  | <115 |
| Always | <50 | 63(11.8%) |  | <110 |
| Missing |  | 2 |  | 2 |
| ***Watched TV shows or videos just before falling asleep, n (%)*** |  |  | 0.2326 |  |
| Never | 43(11.7%) | 64(12%) |  | 107(11.9%) |
| Almost Never | 33(9%) | 57(10.7%) |  | 90(10%) |
| Sometimes | 103(28.1%) | 175(32.9%) |  | 278(31%) |
| Almost Always | <115 | 126(23.7%) |  | <240 |
| Always | <80 | 110(20.7%) |  | <190 |
| Missing | 1 | 3 |  | 4 |
| ***Used a phone, computer, or electronic device just before falling asleep, n (%)*** |  |  | 0.2137 |  |
| Never | 25(6.8%) | 59(11.1%) |  | 84(9.3%) |
| Almost Never | 25(6.8%) | 37(6.9%) |  | 62(6.9%) |
| Sometimes | 72(19.7%) | 114(21.4%) |  | 186(20.7%) |
| Almost Always | 98(26.8%) | 133(25%) |  | 231(25.7%) |
| Always | 146(39.9%) | 190(35.6%) |  | 336(37.4%) |
| Missing | 1 | 2 |  | 3 |
| ***Needed someone with him/her to fall asleep, n (%)*** |  |  | 0.3237 |  |
| Never | 280(76.5%) | 432(80.9%) |  | 712(79.1%) |
| Almost Never | 39(10.7%) | 47(8.8%) |  | 86(9.6%) |
| Sometimes | <30 | 29(5.4%) |  | <60 |
| Almost Always | 8(2.2%) | 16(3%) |  | 24(2.7%) |
| Always | <15 | 10(1.9%) |  | <25 |
| Missing | 1 | 1 |  | 2 |
| ***Followed a bedtime routine before falling asleep, n (%)*** |  |  | 0.0007 |  |
| Never | 107(29.2%) | 113(21.2%) |  | 220(24.5%) |
| Almost Never | 63(17.2%) | 73(13.7%) |  | 136(15.1%) |
| Sometimes | 99(27%) | 134(25.2%) |  | 233(25.9%) |
| Almost Always | 53(14.5%) | 121(22.7%) |  | 174(19.4%) |
| Always | 44(12%) | 91(17.1%) |  | 135(15%) |
| Missing | 1 | 3 |  | 4 |
| ***Woke up at about the same time every day, n (%)*** |  |  | 0.1331 |  |
| Never | 31(8.5%) | 34(6.4%) |  | 65(7.2%) |
| Almost Never | 29(7.9%) | 38(7.1%) |  | 67(7.4%) |
| Sometimes | 123(33.6%) | 150(28.1%) |  | 273(30.3%) |
| Almost Always | <125 | 201(37.6%) |  | <330 |
| Always | <60 | 111(20.8%) |  | <175 |
| Missing | 1 | 1 |  | 2 |
| ***Tried to fall asleep at about the same time every day, n (%)*** |  |  | 0.8090 |  |
| Never | 47(12.8%) | 60(11.3%) |  | 107(11.9%) |
| Almost Never | 51(13.9%) | 80(15%) |  | 131(14.6%) |
| Sometimes | 127(34.7%) | 173(32.5%) |  | 300(33.4%) |
| Almost Always | 92(25.1%) | 138(25.9%) |  | 230(25.6%) |
| Always | 49(13.4%) | 82(15.4%) |  | 131(14.6%) |
|  | 1 | 2 |  | 3 |

1 Medically underserved areas were defined using MUA designations from the Health Resources & Services Administration (Index of Medical Underservice Score of less than or equal to 62.0 indicates a MUA, scores range from 0 to 100).
