# Supplementary material for: Exploring sleep outcomes in youth across settings: Are there differences based on rurality or medically underserved status in the ECHO cohort?
Source: Sleep Med. Author manuscript; Available in PMC 2026 Apr 1. (PMC13043269; doi:10.1016/j.sleep.2025.108754)
Supplement: Supplement File A [file NIHMS2157873-supplement-Supplement_File_A.pdf]

| ECHO Environmental influences on Child Health Outcomes A program supported by the NIH | **Sleep Health of Children and Adolescents 2 - Parent Report**<br>ECHO-wide Cohort Version 01.30 / September 28, 2020 | **Form SHCA2-PR**<br>Page **1** of **4** |
|---|---|---|

| COHORT ID | SITE ID | PARTICIPANT ID | PIN | COHORT VISIT ID | FORM COMPLETED |
|---|---|---|---|---|---|
| __ __ __ __ __ | __ __ __ __ | ____________ | __ __ __ | __________ | __ __/__ __/__ __ __ __<br>*mm dd yyyy* |

| ECHO LIFE STAGE | | RESPONDENT | |
|---|---|---|---|
| ☐01 Prenatal | ☐02 Perinatal | ☐01 Participant | ☐02 Biological Mother |
| ☐03 Infancy | ☐04 Early Childhood | ☐03 Biological Father | ☐04 Other Respondent → Code: __ __ |
| ☐05 Middle Childhood | ☐06 Adolescence | | |

**STUDY STAFF INSTRUCTION: This form should be completed by the child's primary caregiver during the early childhood, middle childhood, and adolescence life stages. Alternatively, children who are 8 years of age or older can complete the *Self Report* version of the form. The child's ID should be used in the header for the participant ID.**

**INSTRUCTIONS**
*This form has 3 sections:*

- *Section A. Children's Sleep Habits, All Ages*
- *Section B. Children's Sleep Quality, Ages 1-4 Years*
- *Section C. Children's Sleep Quality, Ages 5 Years and Older*

*If your child is 1-4 years old, please Complete Sections A and B. If your child is 5 years or older, please complete Sections A and C.*

*Please think about your child's sleep during the past week (past 7 nights) when answering the following questions.*

## Section A. Children's Sleep Habits, All Ages

**1.** How many hours did your child usually spend sleeping **during the NIGHT**?

|___|___| *hours* AND |___|___| *minutes*

**2.** How many days did your child take a daytime nap?

☐00 None → ***Skip to question 4***
☐01 1 day
☐02 2 - 3 days
☐03 4 - 5 days
☐04 6 - 7 days

**3.** When your child did take a daytime nap, how much time did your child usually sleep?

|___|___| *hours* AND |___|___| *minutes*

**4.** What was your child's bedtime on **WEEKDAYS** (Sunday night – Thursday night)?

**WEEKDAY**
*Bedtime:* ___ ___ : ___ ___ ☐01 AM ☐02 PM
*hour minute*

**5.** When did your child usually wake up on **WEEKDAYS** (Monday morning – Friday morning)?

**WEEKDAY**
*Wake-up time:* ___ ___ : ___ ___ ☐01 AM ☐02 PM
*hour minute*

**Section A. Children's Sleep Habits, All Ages (Continued)**

**6.** What was your child's bedtime on **WEEKENDS** (Friday and Saturday nights)?

**WEEKEND**
*Bedtime:* ___ ___ **:** ___ ___ (*hour* / *minute*) ☐01 AM ☐02 PM

**7.** When did your child usually wake up on **WEEKENDS** (Saturday and Sunday mornings)?

**WEEKEND**
*Wake-up time:* ___ ___ **:** ___ ___ (*hour* / *minute*) ☐01 AM ☐02 PM

**8.** In the past 7 days, my child "put-off" or delayed bedtime.

☐01 Never
☐02 Almost Never
☐03 Sometimes
☐04 Almost Always
☐05 Always

**9.** How long did it typically take your child to fall asleep when put to sleep at night? (Example: If your child went to bed at 8:15 PM and your child fell asleep at 8:30 PM, it took 0 hours and 15 minutes for your child to fall asleep.)

|___|___| *hours* AND |___|___| *minutes*

*For the next 3 questions, think about your child's sleep on days when there were no scheduled activities (for example: no school, daycare, work, athletics). These are called **"free" days.***

**10. On "free" days**, what time did your child wake up?

*Wake-up time:* ___ ___ **:** ___ ___ (*hour* / *minute*) ☐01 AM ☐02 PM

**11. On nights before "free" days**, what time did your child go to bed (body in bed)?

*Bedtime:* ___ ___ **:** ___ ___ (*hour* / *minute*) ☐01 AM ☐02 PM

**12. On nights before "free" days**, how much time did it take your child to fall asleep (after the lights were turned out)?

|___|___| *hours* AND |___|___| *minutes*

**13.** For survey routing purposes, how old is your child?

☐01 1-4 years old → ***Skip to Section B, Question 1***
☐02 5 years old or older → ***Skip to Section C, Question 1***

**Section B. Children's Sleep Quality, Ages 1-4 Years**

Please respond to each question or statement by marking one box per row.

| **In the past 7 days…** | **Never** | **Almost Never** | **Sometimes** | **Almost Always** | **Always** |
|---|---|---|---|---|---|
| **1.** My child woke up in the morning feeling rested and alert. | ☐05 | ☐04 | ☐03 | ☐02 | ☐01 |
| **2.** My child snored during sleep. | ☐01 | ☐02 | ☐03 | ☐04 | ☐05 |
| **3.** My child got enough sleep. | ☐05 | ☐04 | ☐03 | ☐02 | ☐01 |
| **4.** My child woke up at night and had trouble falling back to sleep. | ☐01 | ☐02 | ☐03 | ☐04 | ☐05 |
| **5.** My child had difficulty falling asleep. | ☐01 | ☐02 | ☐03 | ☐04 | ☐05 |
| **6.** My child tossed and turned at night. | ☐01 | ☐02 | ☐03 | ☐04 | ☐05 |
| **7.** My child had trouble sleeping. | ☐01 | ☐02 | ☐03 | ☐04 | ☐05 |

**Section C. Children's Sleep Quality, Ages 5 Years and Older**

Please respond to each question or statement by marking one box per row.

| **In the past 7 days…** | **Never** | **Almost Never** | **Sometimes** | **Almost Always** | **Always** |
|---|---|---|---|---|---|
| **1.** My child woke up in the morning feeling rested and alert. | ☐05 | ☐04 | ☐03 | ☐02 | ☐01 |
| **2.** My child snored during sleep. | ☐01 | ☐02 | ☐03 | ☐04 | ☐05 |
| **3.** My child got enough sleep. | ☐05 | ☐04 | ☐03 | ☐02 | ☐01 |
| **4.** My child woke up at night and had trouble falling back to sleep. | ☐01 | ☐02 | ☐03 | ☐04 | ☐05 |
| **5.** My child had difficulty falling asleep. | ☐01 | ☐02 | ☐03 | ☐04 | ☐05 |
| **6.** My child slept through the night. | ☐05 | ☐04 | ☐03 | ☐02 | ☐01 |
| **7.** My child had a problem with his/her sleep. | ☐01 | ☐02 | ☐03 | ☐04 | ☐05 |
| **8.** My child was sleepy during the daytime. | ☐01 | ☐02 | ☐03 | ☐04 | ☐05 |
| **9.** My child had a hard time concentrating because he/she was sleepy. | ☐01 | ☐02 | ☐03 | ☐04 | ☐05 |
| **10.** My child had a hard time getting things done because he/she was sleepy. | ☐01 | ☐02 | ☐03 | ☐04 | ☐05 |
| **11.** My child had problems during the day because of poor sleep. | ☐01 | ☐02 | ☐03 | ☐04 | ☐05 |
| **12.** My child had trouble sleeping. | ☐01 | ☐02 | ☐03 | ☐04 | ☐05 |

| **Setting** | **Mode** |
|---|---|
| ☐01 Clinic or site ☐02 Phone ☐03 Other location | ☐01 Self-administered ☐02 Staff-administered |

PROMIS Early Childhood Parent Report v1.0 - Sleep Health Short Form 4b. © 2008-2018 PROMIS Health Organization and PROMIS Cooperative Group.
PROMIS Parent Proxy v1.0 Sleep Disturbance Short Form 4a. © 2008-2018 PROMIS Health Organization and PROMIS Cooperative Group.
PROMIS Parent Proxy v1.0 Sleep-Related Impairments Short Form 4a © 2008-2018 PROMIS Health Organization and PROMIS Cooperative Group.
